# Serratus anterior palsy as a Masquerader: utilizing novel digital dynamic radiography for diagnosis and treatment response

**DOI:** 10.1016/j.jseint.2023.12.001

**Published:** 2024-01-11

**Authors:** Sameer R. Khawaja, John M. Kopriva, Zaamin B. Hussain, Hayden L. Cooke, Michael B. Gottschalk, Eric R. Wagner

**Affiliations:** Department of Orthopaedic Surgery, Emory University School of Medicine, Atlanta, GA, USA

**Keywords:** Serratus anterior palsy, Dynamic digital radiography, Pectoralis major transfer, Scapulohumeral rhythm

Scapulothoracic motion is dependent on a force couple balance between the various periscapular muscles, enabling scapular internal and external rotation, protraction and retraction, and medial and lateral tilt.^1^ Dysfunction of any of the muscles can result in scapulothoracic abnormal motion, leading to an abnormal scapulohumeral rhythm (SHR) and motion of the shoulder complex.^20^ Scapular winging is one type of scapulothoracic abnormal motion, caused by either traumatic injury or chronic compression of either the long thoracic nerve (LTN) or spinal accessory nerve (innervating the serratus anterior (SA) and trapezius muscles, respectively).^20^^,^^27^ The SA plays a critical role in scapular stabilization against the chest wall, as well as scapula external rotation by pulling the scapula anterolaterally around the thorax, thereby elevating the acromion, enabling abduction and elevation of the glenohumeral joint.^18^ It acts as in a force couple with the rhomboids and levator scapulae in stabilizing the scapula during SHR. Therefore, a palsy or atrophy of the SA causes an internally rotated and medially prominent resting position due to unopposed pull of the rhomboid major and minor and levator scapulae; which is worsened with attempted arm elevation.^8^

The incidence of an isolated SA palsy due to LTN compression is rare, 0.0026% for an isolated paralysis,^24^ but can be severely disabling. In cases that do not spontaneously resolve, surgical interventions, such as the sternal head of the pectoralis major transfer can be considered.^3^^,^^6^^,^^8^ This transfer involves transferring the upper and lower sternal heads pectoralis major from its insertion on the humerus to the inferior angle of the scapula, recreating a force vector of the SA that stabilizes the scapula on the chest wall.

The diagnosis of SA palsy is challenging as the presentation is often nonspecific shoulder pain and limited range of motion (ROM). Physical examination involves inspection of a medially-prominent internally rotated scapular resting posture, as well as a lack of scapular protraction against resistance, and a positive shoulder flexion resistance test and scapular compression test.^12^^,^^17^^,^^21^^,^^22^^,^^29^ However, these are often difficult to perform and ability to partake in examination can be limited by impingement pain or instability from a lack of normal SHR.^23^^,^^30^ Magnetic resonance imaging and standard radiographs may be normal as these are static investigations of a dynamic process. Electromyogram (EMG) testing aids in the diagnosis, but testing the SA is technically difficult and technique dependent, and clinicians hesitate to rely solely on EMG findings for indicating surgery.^10^^,^^24^^,^^26^

To overcome these challenges, Dynamic Digital Radiography (DDR) has emerged as a promising tool for in-office imaging of dynamic shoulder pathologies such as rotator cuff tears, anterior and posterior shoulder instability, and adhesive capsulitis.^14^^,^^33^ DDR allows for an objective measurement of SHR - the ratio between the change in humeral motion and the change in scapula motion during arm abduction, using standardized positioning and protocol.^14^^,^^33^ DDR empowers the surgeon to make an in-office diagnosis to more appropriately guide further work-up or surgical interventions, and aids with patient communication, education, and compliance with therapy. Furthermore, DDR can be used to evaluate response to treatment.

In this case report, we aim to present a case of the novel split pectoralis major transfer to treat SA paralysis, using DDR to objectively demonstrate the postoperative improvement in scapulothoracic motion and SHR.

## Case report

### Patient presentation and physical examination

A 55-year-old right-hand dominant woman presented with debilitating right shoulder pain and dysfunction due to scapular winging. Her previous medical history was notable for a cervical fusion, rotator cuff repair on the right shoulder prior to presentation, and a right LTN decompression four years prior to presentation. Pain and function were recalcitrant to these interventions, with pain rated 8 out of 10, limitations in shoulder ROM and a shoulder subjective value of only 40%.

Physical examination demonstrated scapular winging, with atrophy of the right SA muscle, and a medially prominent and internally rotated resting posture. The shoulder flexion resistance test and scapular protraction test both indicated SA dysfunction. Scapular stabilization testing resulted in marked improvement in shoulder motion. Attempted shoulder elevation and abduction showed scapular asymmetry, with more pronounced medial prominence and scapular internal rotation. ROM of the affected shoulder was limited to active flexion of 100 degrees and active abduction of 80 degrees. With elbows at her side, external rotation was 60 degrees (active). The patient could reach L1 vertebral body on internal rotation.

**Figure 1:**
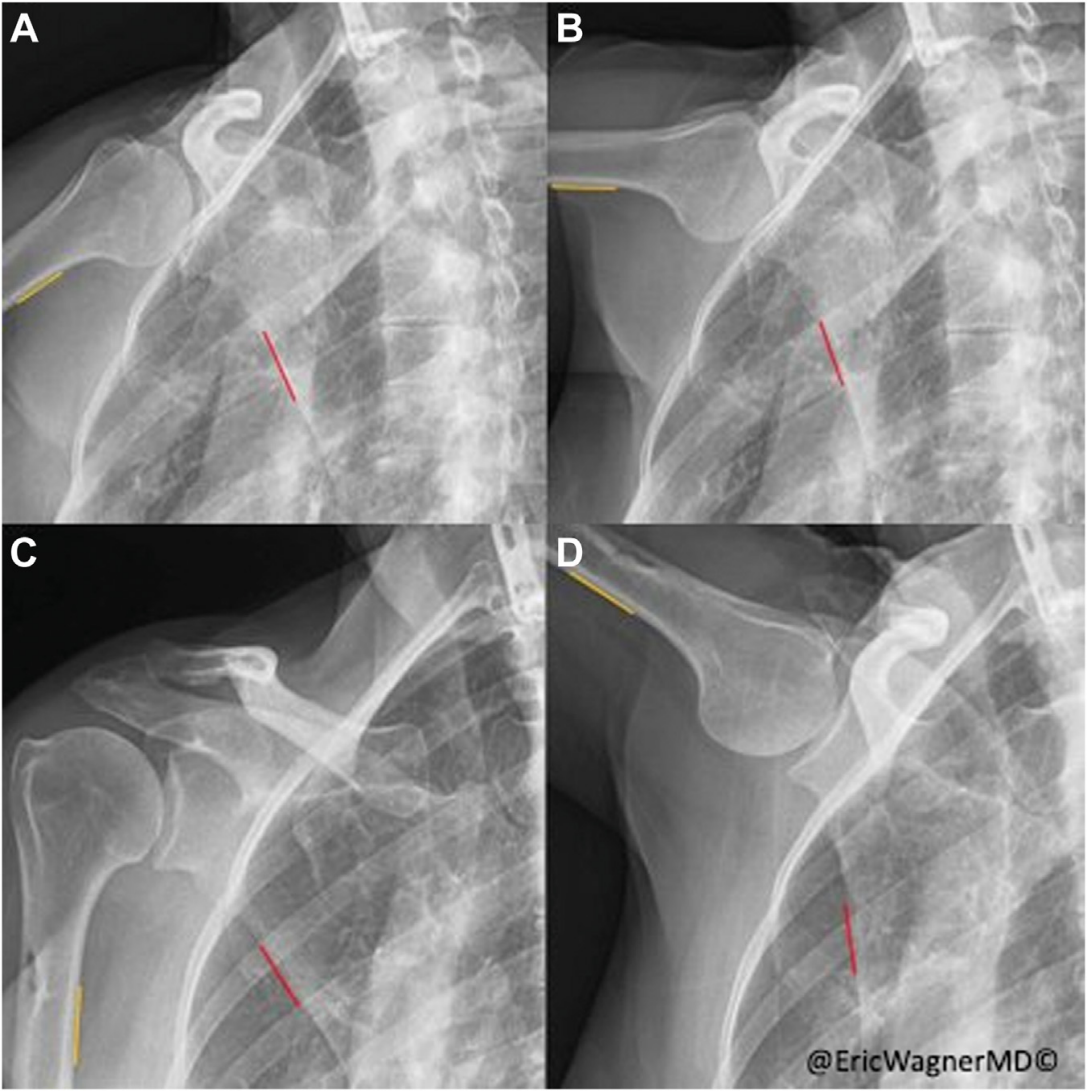
Clips taken from preoperative (**A**, **B**) and postoperative (**C**, **D**) DDRs displaying minimal (**A**, **C**) and maximal (**B**, **D**) angles of humeral abduction. Yellow lines indicate the zone used to measure humerothoacic angle. Red lines indicate the zone used to measure scapulothoracic angle. **A** and **B** show minimal to no change in scapulothoracic angle while **C** and **D** demonstrate a marked change in the scapulothoracic angle due to the restored external rotation of the scapula in **D**. *DDR*, dynamic digital radiography.

In-office DDR of the right shoulder demonstrated arm abduction limitations and a dysfunctional SHR, the ratio between the change in humerothoracic angle over the scapulothoracic angle, calculated to be 7.8 through her limited arm abduction from 45° to 90° (Fig. 1 *A* and *B*) (Video 1). The EMG also demonstrated limited conduction through the LTN. Together with the DDR and EMG, the patient was diagnosed with a SA palsy.

### Surgical technique

**Figure 2:**
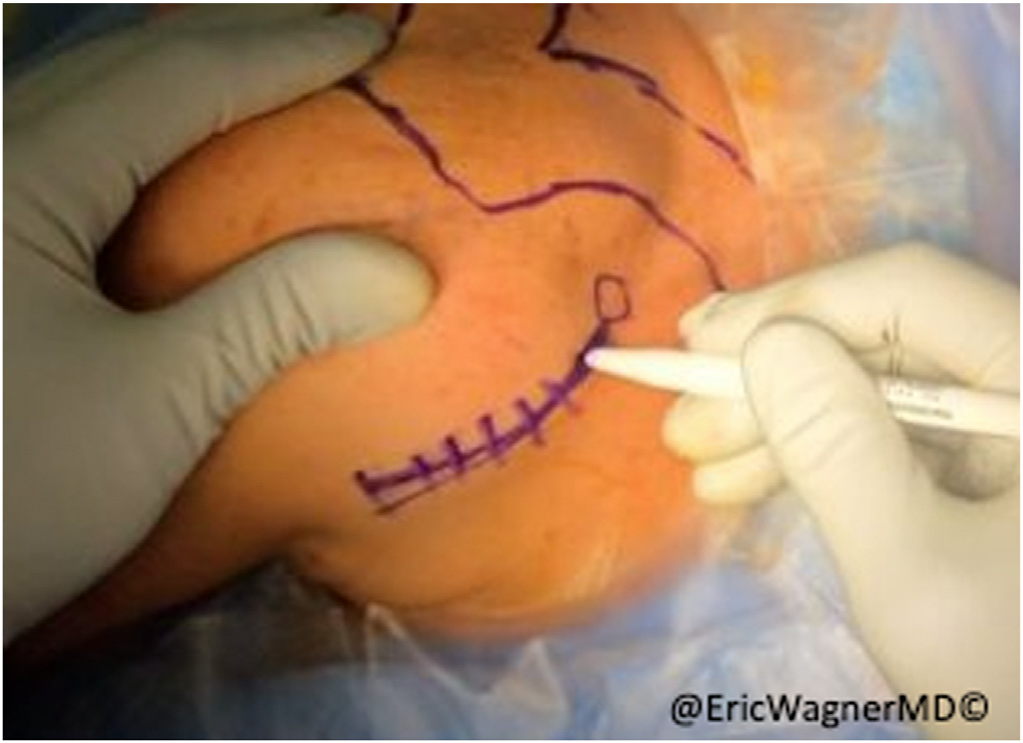
Modified deltopectoral approach.

**Figure 3:**
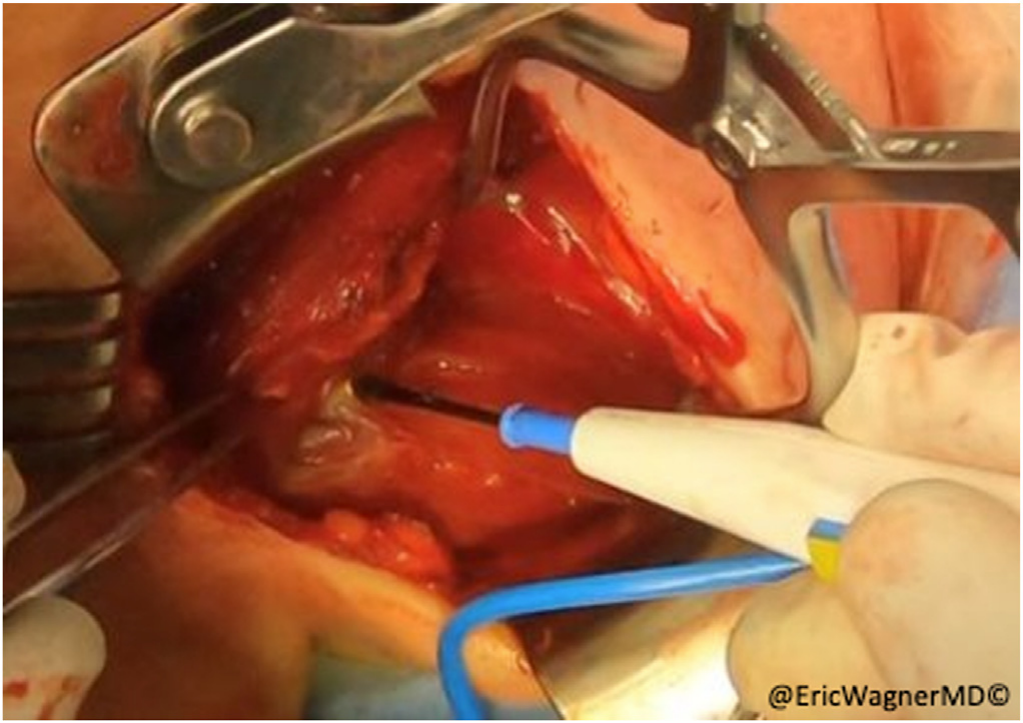
A fat stripe between the clavicular and sternal heads of the pectoralis major is identified and the interval is developed.

**Figure 4:**
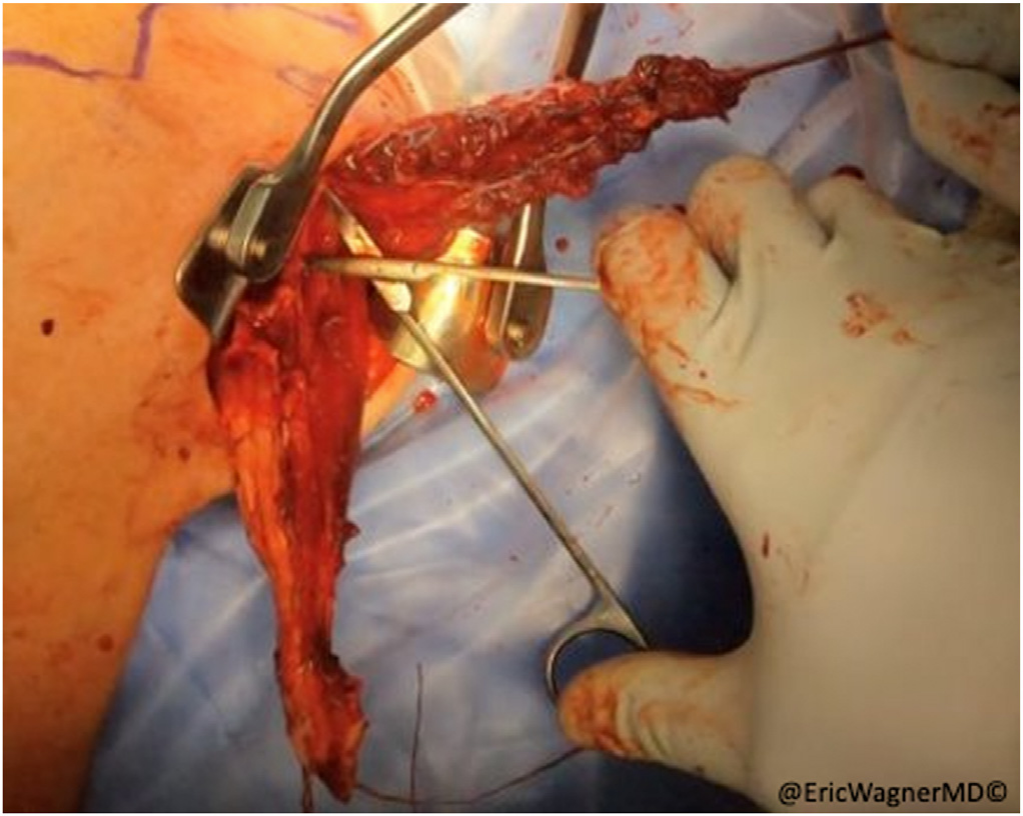
With the clavicular and sternal heads of the pectoralis major separated, krakow sutures are placed.

After failure of non-surgical interventions, the patient underwent a sternal head of the pectoralis major transfer. In this procedure, the insertion of the sternal head of the pectoralis major was carefully dissected, isolated and harvested with a bone plug from the proximal humerus and transferred to the lateral edge of the inferior angle of the scapula, per the technique of Elhassan et al.^8^ This technique involves exposing the pectoralis major through a modified deltopectoral approach (Fig. 2). After opening the deltopectoral interval, the pectoralis is exposed, including the inferior and superior aspects of the muscle. The interval between the clavicular head and sternal heads is identified with a fat stripe pointing in the direction of the sternoclavicular joint (Fig. 3). The clavicular head’s smaller tendon wraps over the top of the larger sternal head tendon as they insert onto the humerus. Electrocautery elevates the clavicular head from the humerus and tenotomies are used to further develop the interval with the sternal head. A krackow stitch with #2 non-absorbable suture is placed for later repair. The sternal head’s insertion is then osteotomized, using a small burr or drill, then connecting the holes with a sagittal saw. Another krackow stitch with #2 non-absorbable suture is placed. The two heads are then further separated (Fig. 4).

**Figure 5:**
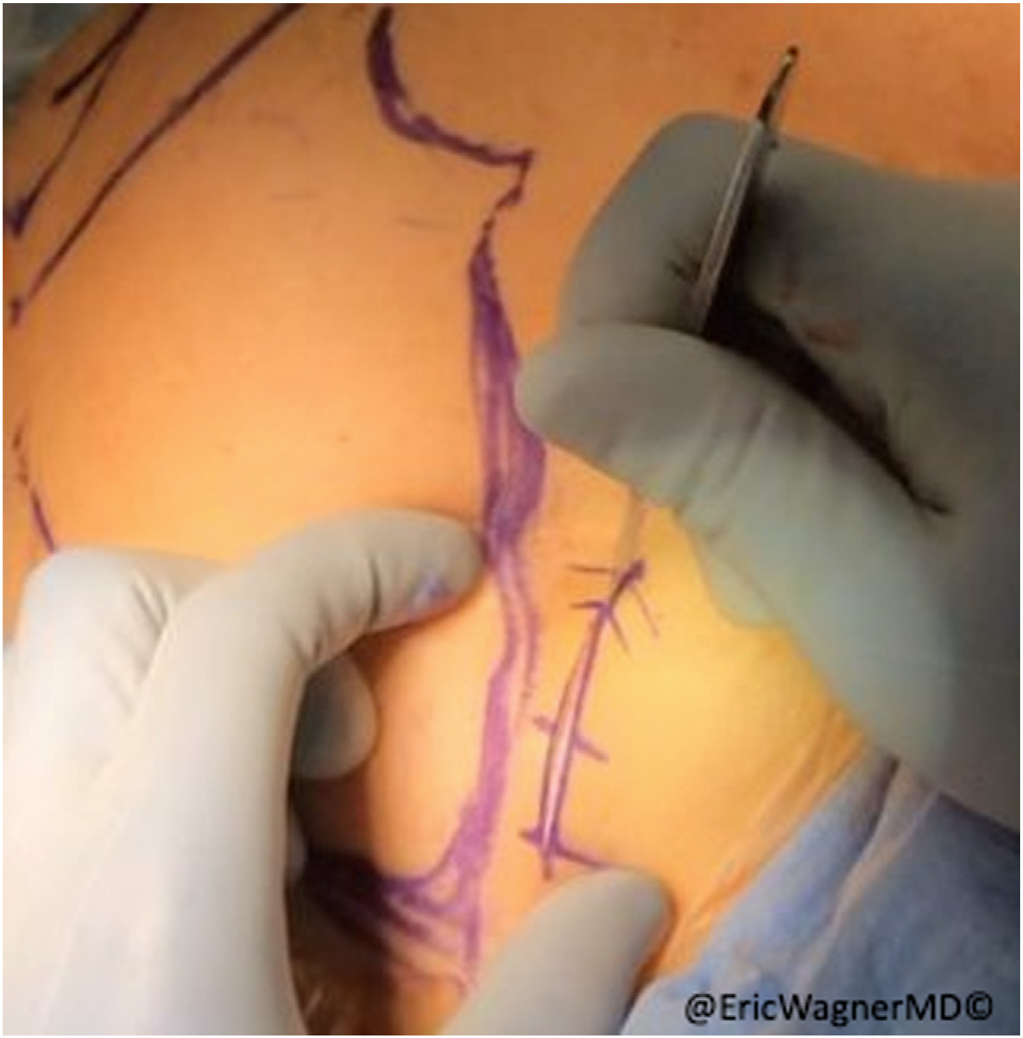
Approaching the scapula at the inferior angle just lateral to the inferolateral border of the scapula.

**Figure 6:**
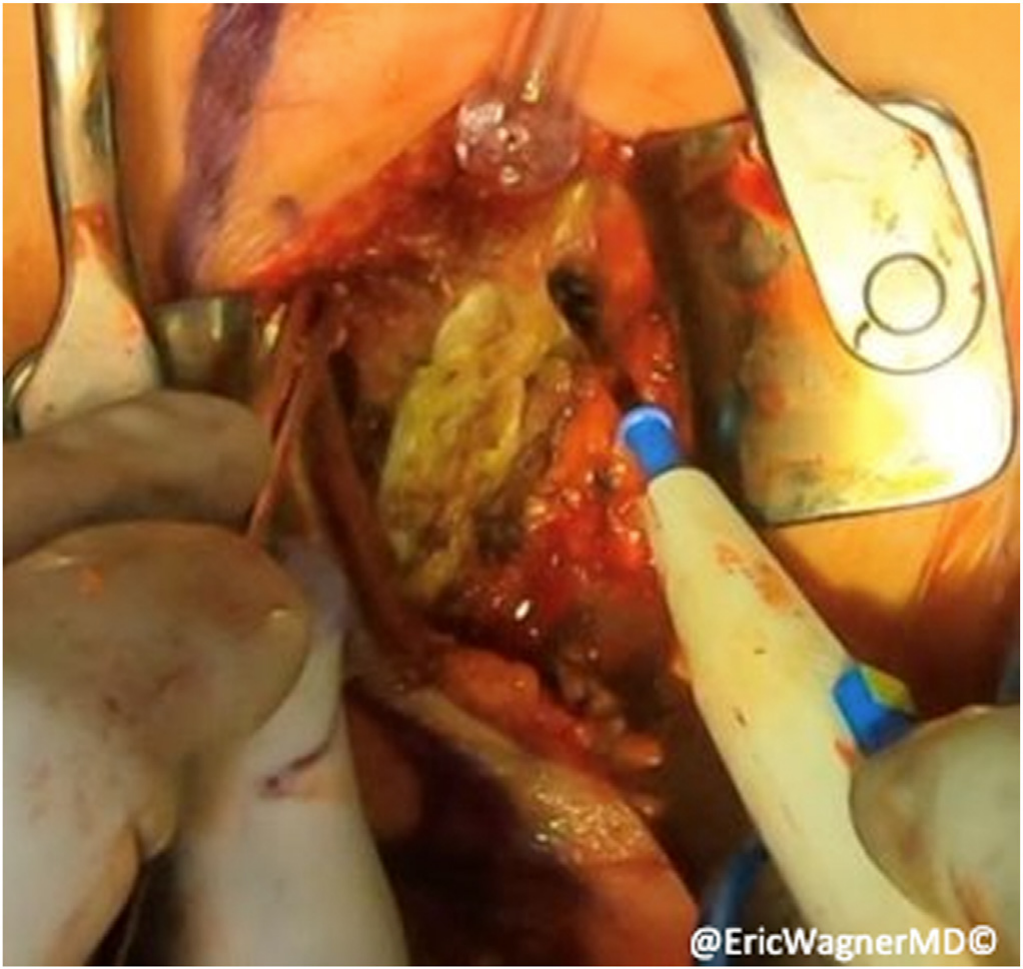
Exposed inferior angle of scapula, partially peeling the teres major origin.

**Figure 7:**
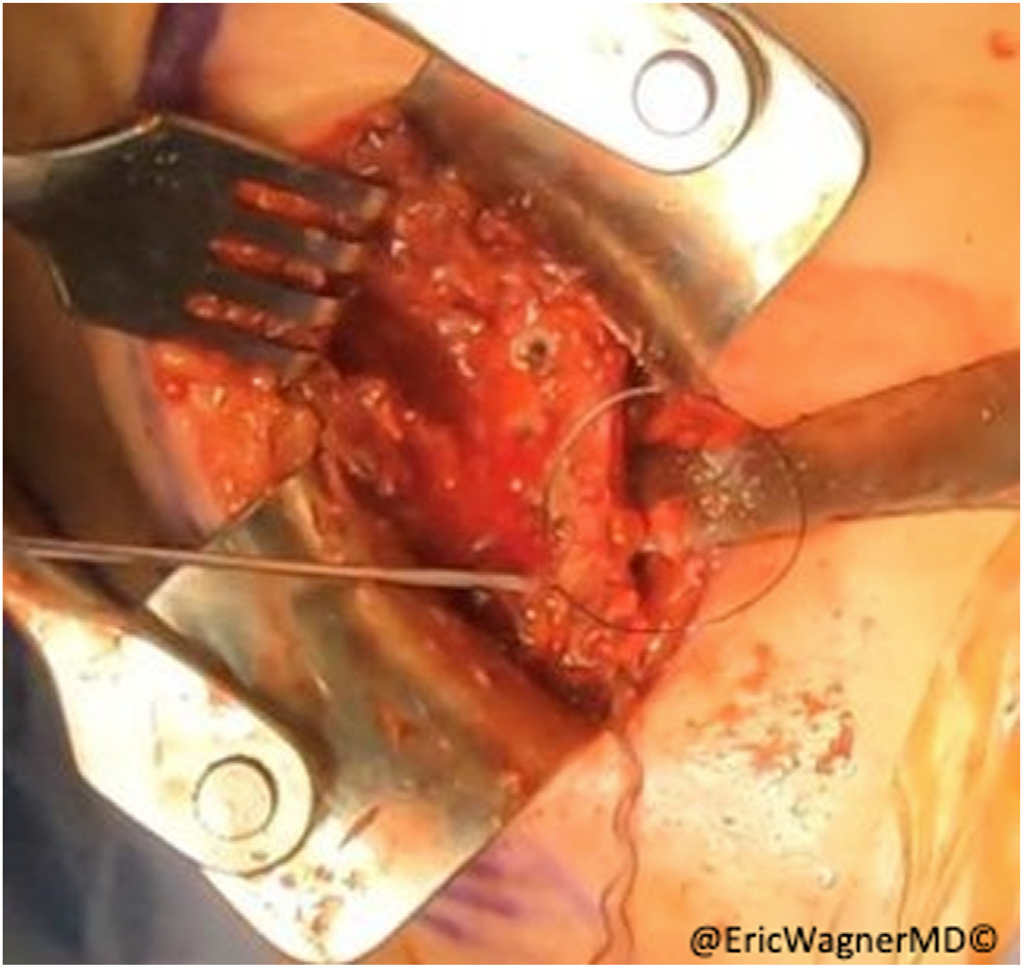
Inferior angle of scapula with burr holes prior to passing of graft.

**Figure 8:**
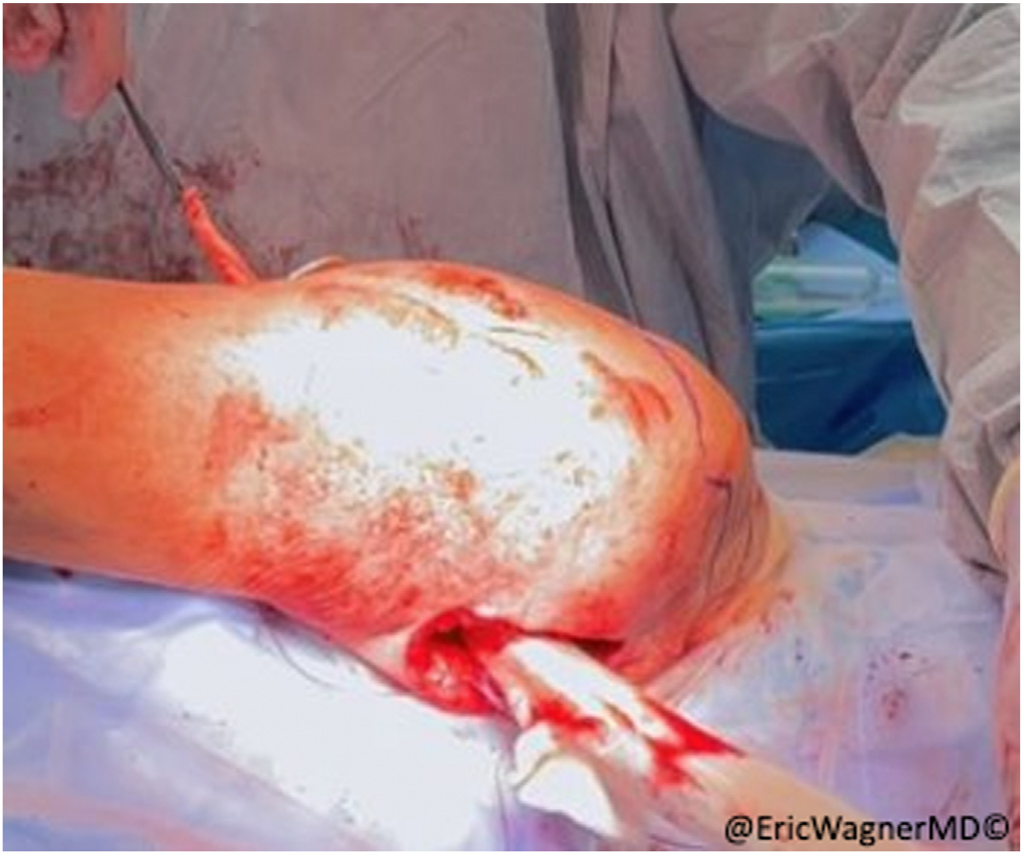
After bluntly creating the path along the chest wall between the anterior and posterior incisions, a lap sponge may be slid back and forth to enlarge the track.

**Figure 9:**
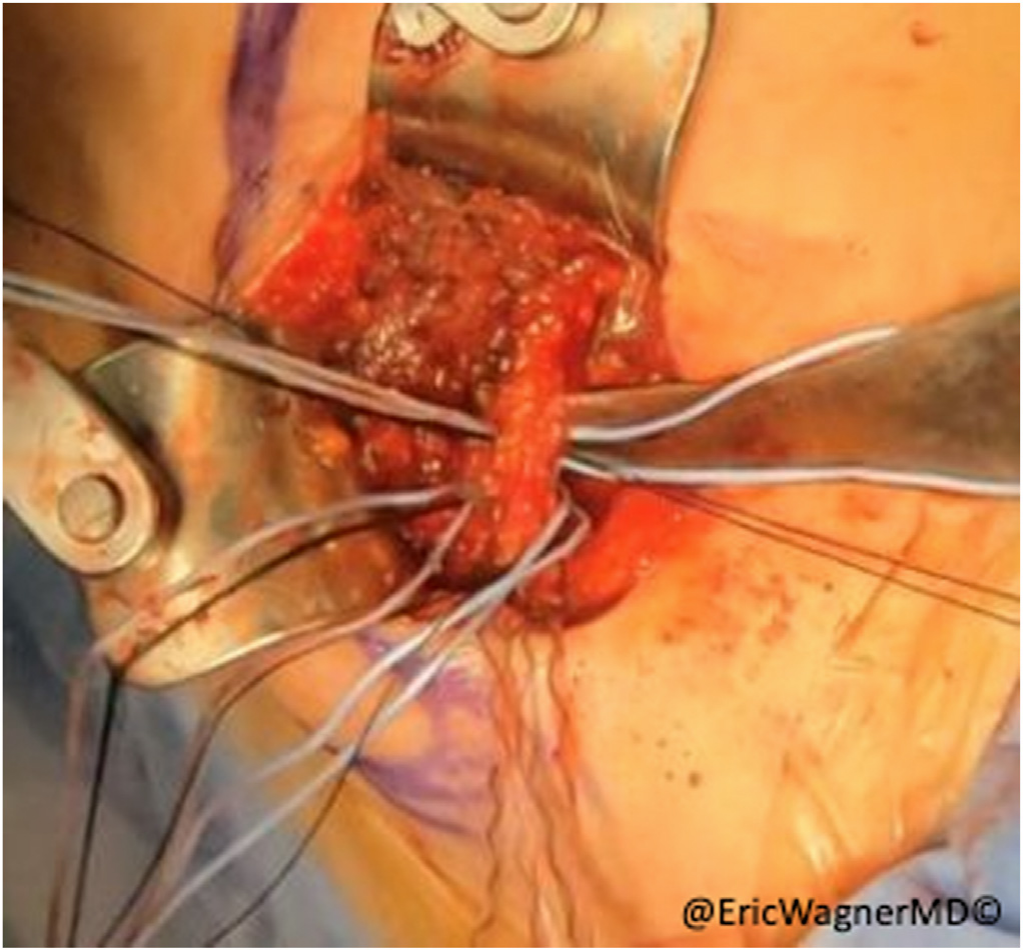
With the sternal head of the pec tendon transferred (including its osseous insertion from the humerus), transosseous sutures with #2 non-absorbable sutures are looped through the drill holes in the scapula and around the osseous portion of the transfer.

**Figure 10:**
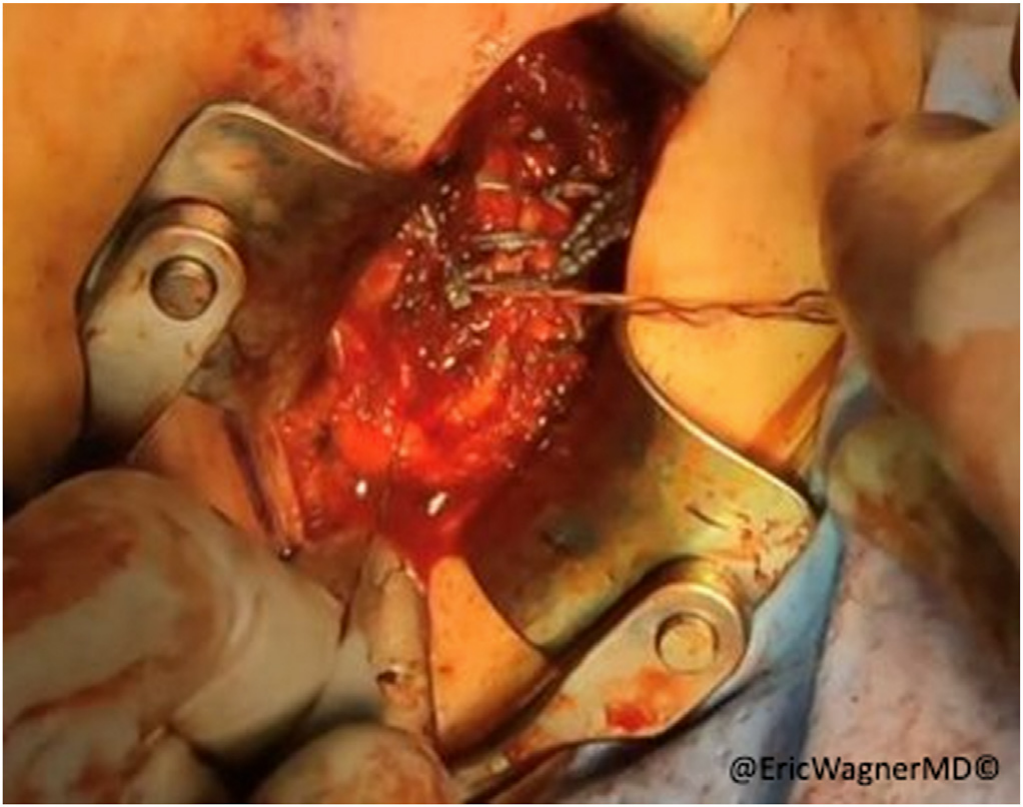
Modified Nice knot to secure osseous insertion of sternal head to the lateral edge of the inferior scapula.

The inferior aspect of the scapula is approached using a diagonal incision just lateral to is lateral edge (Fig. 5). After inferiorly retracting the latissimus dorsi, the teres major is identified originating from the inferior lateral edge of the scapula (Fig. 6). The remnant SA tendon is tagged with #2 non-absorbable sutures, and the inferior angle and lateral edge of the scapula is exposed by removing some of the teres major origins (Fig. 7). The transfer is then performed bluntly creating a passage along the chest wall between the anterior and posterior incisions. A lap sponge can be used to enlarge to passageway (Fig. 8). The sternal head is then transferred to the posterior incision in proximity to the scapula. Transosseous sutures (4-5 in total) are placed using #2 non-absorbable sutures in a looped fashion (Fig. 9). The osseous insertion of the sternal head is then secured with the transosseous sutures to the lateral edge of the inferior scapula using a modified Nice knot technique (Fig. 10). The serratus tendon is then also anchored to the sternal head and a normal layered closure is performed. The patient is immobilized for 6 weeks before starting physical therapy.

### Postoperative course

**Figure 11:**
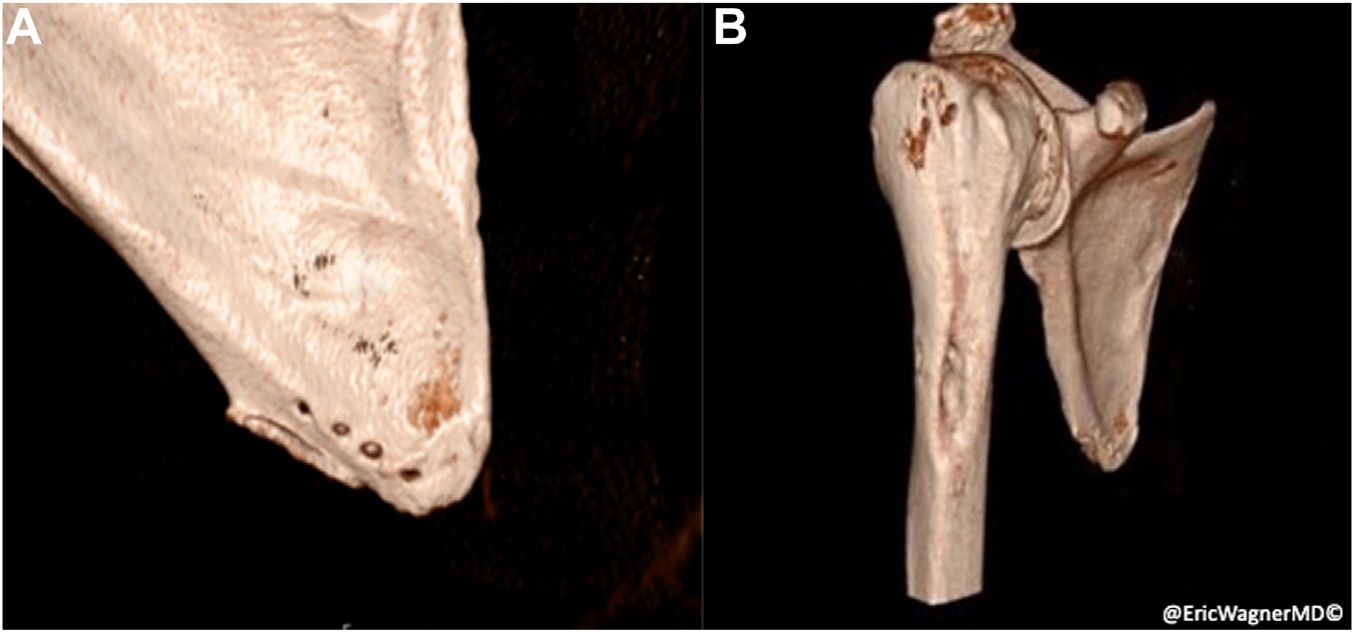
3D reconstructions from 7 months postoperative CT demonstrating osteointegrated humeral bone plug secured with non-absorbable suture looped through transosseous tunnels (**A**) and donor site at pectoralis major insertion (**B**). *CT*, computed tomography.

Following the procedure, the patient displayed improved ROM, strength and endorsed improvement in her pain. Computed tomography at 7 months postoperative demonstrated a well corticated humeral donor site from the pectoralis harvest and osseous healing at its new insertion on the inferior angle of the scapula (Fig. 11).

Of note, SHR is typically calculated across the full arc of motion and at standard intervals of 0-30, 30-60, and 60-90.^14^ Due to this patient’s limited preoperative ROM from 45° to 90° of humeral abduction (Fig. 1 *A* and *B*), we calculated SHR (total arc) to be 7.8. DDR obtained 10 months postoperative demonstrated significant improvements in active arm abduction to 120° (Fig. 1 *C* and *D*) (Video 1). The SHR across her improved full range of abduction (0°-120°) was 3.0 and across the interval of 45°-90° (her preoperative arc) was 2.7 (from 7.8). This is consistent with a normal SHR in an uninjured shoulder.^15^^,^^28^ These measurements provide objective data to support the clinically observed improvements in shoulder function—in range and control of motion.

## Discussion

Scapular motion is complex but integral to a functional shoulder. The periscapular muscles, including the SA, play a crucial role in scapulothoracic motion,^30^ as the SA stabilizes the scapula to allow for protraction.^18^ With the loss of the SA, other periscapular muscles, such as the levator scapulae and rhomboids, cause scapular internal rotation, distal pole retraction, and medial prominence.^20^ Diagnosing SA palsy is difficult as the clinical features mimic other common shoulder pathologies, leading to incorrect or delayed diagnoses, unnecessary operations, and delays in the correct treatment course with subsequent worsened outcomes.^30^ Currently, EMG testing is the only definitive diagnostic test for SA palsy,^9^^,^^10^^,^^16^^,^^30^ but not all patients with scapular winging secondary to SA palsy show EMG nerve dysfunction.^30^^,^^32^ DDR is a novel radiographic imaging technique that captures pulsed low-radiation shoulder radiographs, allowing for the assessment of dynamic scapulothoracic motion.^33^ DDR can be implemented into clinical workflow to diagnose scapular pathologies, such as SA palsy, by objectively measuring parameters such as SHR to improve diagnosis and treatment of scapular winging.^14^^,^^23^

This case reports shows that DDR was not only able to diagnose this patient with SA palsy but also demonstrate restoration of scapular biomechanics, and success of the split pec-major tendon transfer. SHR for a normal shoulder has been reported as 2-3,^15^^,^^28^ varying during different degrees of humeral abduction.^5^^,^^7^^,^^25^^,^^28^^,^^31^^,^^33^ DDR eloquently demonstrates that scapulothoracic dysfunction and associated compensation at the glenohumeral joint to obtain humerothoracic elevation, can result in drastically increased SHR values in an attempt to achieve arm abduction. Close to normal SHR was obtained and relatively normal biomechanics were restored following a split-pectoralis major tendon transfer, supporting our diagnosis and selection of the appropriate intervention. Furthermore, this technology demonstrates the effectiveness of using the split pectoralis major transfer to restore close to normal scapulohumeral kinematics. Elhassan et al were the first to show excellent outcomes with pectoralis major transfer for SA dysfunction, citing the avoidance of a tendon graft to reach the scapula and direct bone-to-bone healing at the site of transfer as major advantages of the procedure.^8^ The vast majority of patients reported complete resolution of scapular winging and pain and significant improvements in shoulder ROM. Mean shoulder Constant score significantly improved from 49 preoperatively to 82 posteropatively, while mean shoulder subjective value significantly improved from 60% to 84%.^8^

Historically, SHR can be difficult, time-consuming, and inaccurate to determine in the clinic. SHR has traditionally been measured using goniometry,^2^ inclinometers,^28^ and skin or electromagnetic sensors,^2^^,^^4^^,^^13^ all of which are susceptible to error.^4^^,^^28^^,^^33^ In-plane dynamic radiographic analysis allows for accurate measurements of SHR,^19^ but has traditionally required a specialized lab with fluoroscopy. In-office DDR overcomes these limitations and administers similar ionizing radiation exposure as a standard 2-view shoulder radiograph exam. Current literature has already examined dynamic radiography to diagnose shoulder pathologies such as adhesive capsulitis, glenohumeral osteoarthritis, rotator cuff tears, and posterior shoulder instability.^14^^,^^33^ Xiao et al used DDR to suggest that the SHR of normal shoulders may actually be closer to 3.5 for complete shoulder motion.^33^ However, this was using the medial scapula border for measurement analysis which is thought to overestimate the SHR. Additionally, using a dynamic biplane fluoroscopy system and computed tomography 3D position to match the position of the scapula, Giphart et al found the SHR to be 2.0 ± 0.4:1 for abduction.^11^ Therefore, restoring SHR from 7.8 to 2.7 represents a major improvement in scapula biomechanics, to relatively normal level, providing objective data of the surgical success.

Dynamic radiography has limitations that must be addressed before it can be used to diagnose SA palsy. While each patient is presented with verbal cues and coaching from radiology technicians on cadence and effort during capture of a DDR, some patients may lean over or not give maximal effort, limiting maximal abduction values. Although dynamic and more precise than physical exam estimates, SHR measurements in DDR are two-dimensional, while the motion of the scapula is three-dimensional. Future developments may introduce measurements in orthogonal views. As the literature on DDR expands, it can be implemented into orthopedic clinical workflow as a time-efficient, cost-effective modality to diagnose shoulder pathologies and the response to treatment with limited radiation exposure.

## Conclusion

The use of DDR can be used to assist with the diagnosis of SA palsy and other challenging scapular patholigies. Furthermore, it can help track improvements in shoulder function and fluidity following surgical intervention. Establishing DDR in the clinical workflow can reengage this measurement as a valuable tool in diagnosing complex and surgical shoulder pathologies. Furthermore, the use of a split pectoralis major transfer to treat SA paralysis is able to restore normal scapulohumeral kinematics. This case report is a novel example displaying the versatility of DDR in clinical workflow, both as a diagnostic modality and way to monitor postoperative improvement.

## Disclaimers

Funding: No funding was disclosed by the authors.

Conflicts of interest: Eric R. Wagner is a consultant for Stryker Corporation, Acumed, Osteoremedies, and Zimmer Biomet. He also receives research institutional research support from Arthrex and Konica Minolta. Michael B. Gottschalk receives research support from Stryker Corporation, Konica Minolta and Arthrex. The other authors, their immediate families, and any research foundation with which they are affiliated have not received any financial payments or other benefits from any commercial entity related to the subject of this article.

Patient consent: The patient in this case was informed on the content of and utilization of this report. The patient was informed that confidentiality would be maintained, and the patient provided informed consent for this information to be published.

## Supplementary data

Video 1 Preoperative DDR showing minimal ROM with little scapular involvement for shoulder motion. Postoperative DDR at 10-month follow-up showing complete ROM and improved scapular involvement for shoulder function.
